# THE RELEVANCE OF GENERAL FLUID ABILITIES IN TECHNOLOGY PROFICIENCY IN OLDER ADULTS

**DOI:** 10.1093/geroni/igae098.2120

**Published:** 2024-12-31

**Authors:** Shenghao Zhang, Walter Boot

**Affiliations:** Weill Cornell Medicine, New York, New York, United States; Weill Cornell Medicine, New York, New York, United States

## Abstract

Previous research showed that general fluid abilities are correlated with technology use, acceptance, and proficiency in older adults. Cognitive decline was constantly proposed as an explanation for digital divide and age-related declines in technology use. However, this explanation has not been examined directly. We tested the relevance of general fluid abilities on technology proficiency in two large samples of normally aging older adults cross-sectionally and longitudinally with structural equation modeling. Results from a cross-sectional sample of older adults with various previous technology experience showed that older adults with higher general fluid abilities (measured by a latent factor consisted of letter set and immediate and delayed recall) also had higher technology proficiency (measured by a latent factor consisted of computer proficiency and mobile device proficiency). However, general fluid abilities were not predictive of technology proficiency after controlling for age, and general fluid abilities did not mediate the relationship between age and technology proficiency. Results from a longitudinal sample in which older adults with little to no previous experience with computers were trained to use and used a computer for 12 months showed that general fluid abilities (measured by a latent factor consisted of measures on processing speed, executive function, working memory, and episodic memory) was not predictive of the baseline or the slope of change of computer proficiency. However, those who are older increased less in proficiency controlling for fluid abilities. Discussion will focus on alternative explanations on age-related digital divide.

